# Single-Dose Calcium Channel Blocker Toxicity in a Patient With Severe Liver Disease

**DOI:** 10.7759/cureus.66308

**Published:** 2024-08-06

**Authors:** Shweta Lodha, Daniel Loriaux, Amanda L Faulkner, Kathyrn Pearson, Shreyansh Shah

**Affiliations:** 1 Department of Internal Medicine, Duke University School of Medicine, Durham, USA; 2 Department of Internal Medicine, Duke University Medical Center, Durham, USA; 3 Department of Anesthesiology, Duke University Medical Center, Durham, USA; 4 Department of Neurology, Duke University Medical Center, Durham, USA

**Keywords:** drug toxicity, acoustic neuroma resection, calcium channel blocker, calcium channel blocker toxicity, nimodipine, acoustic neuroma

## Abstract

Calcium channel blockers (CCBs) are commonly used in the management of multiple diseases, including hypertension, arrhythmia, and vasospastic disorder. Nimodipine, a dihydropyridine CCB, has demonstrated utility in preserving hearing following vestibular schwannoma resection surgery. Due to its widespread use, CCB overdose is common. This case report presents a unique case of CCB toxicity in a 56-year-old female with end-stage liver dysfunction. The patient developed vasodilatory shock after receiving a single dose of prophylactic nimodipine following vestibular schwannoma surgery. The primary objective of this report is to highlight the unique risk for CCB toxicity that exists for patients with advanced liver disease who receive nimodipine in the perioperative setting.

## Introduction

Calcium channel blockers (CCBs) constitute a class of medications that antagonize L-type voltage-dependent calcium channels in specialized conduction systems within the myocardium and vascular smooth muscle [1]. CCBs are used in the management of multiple diseases, including hypertension, arrhythmia, angina, and vasospastic disorders [1]. Moreover, nimodipine, a type of dihydropyridine CCB, has demonstrated efficacy in preserving hearing function following vestibular schwannoma and maxillofacial surgery [2].

CCBs are consistently one of the 10 most commonly prescribed medications in North America [3]. CCB toxicity is common, given its widespread use; however, the substantial impacts of CCB toxicity are often underappreciated [1]. Per the American Association of Poison Control, CCBs have the highest degree of mortality due to exposure out of all cardiovascular drugs [4]. Moreover, the widespread use of CCBs has resulted in increased intentional and accidental overdoses [1].

Early identification of patients who are at the highest risk for CCB toxicity is essential for guiding dose adjustments, avoiding drug-drug interactions that can delay metabolism, and facilitating early intervention when signs of CCB toxicity are present. As both dihydropyridine and non-dihydropyridine CCBs undergo metabolism via the cytochrome-P450 (CYP450) system, a collection of enzymes found primarily in the liver that facilitate drug metabolism, patients with severe liver disease constitute a particularly high-risk group of patients for CCB toxicity [5,6]. In this report, we present a case of a patient with advanced cirrhosis who developed CCB toxicity after receiving a single standard dose of prophylactic nimodipine following an uncomplicated vestibular schwannoma resection.

## Case presentation

A 56-year-old female with a past medical history notable for hypertension, stage 2 chronic kidney disease with a baseline Cr of 1.0 and GFR of 66, and alcoholic cirrhosis with a MELD-Na of 21 and portal hypertension presented to the hospital for elective resection of a unilateral vestibular schwannoma. Preoperatively, the patient received a 40 mg tablet of aprepitant for postoperative nausea and vomiting alleviation. In the operating room, the patient received succinylcholine and propofol for rapid sequence intubation. Propofol (60-125 mcg/kg/min) and remifentanil (0.2 mcg/kg/min) were continued for maintenance of general anesthesia.

**Figure 1:**
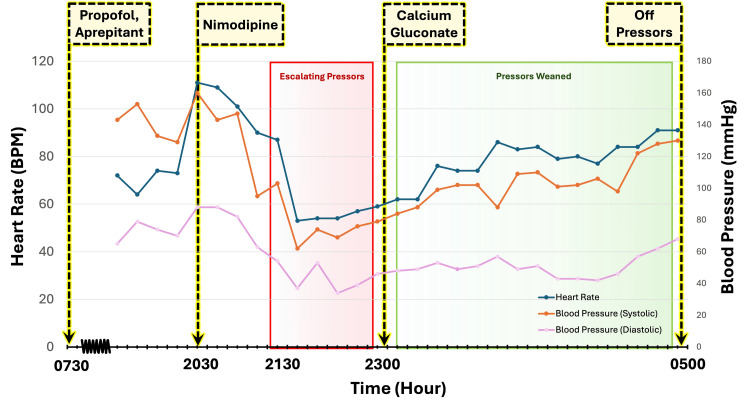
Progression of blood pressure and heart rate on hospital day 1 Trend lines show changes in the patient’s blood pressure and heart rate over the course of hospital day 1, and time points indicate when anesthetic agents, nimodipine, pressors, and calcium gluconate were initiated and/or weaned off.

The surgery lasted for 7.5 hours, and there were no intraoperative complications. The patient was extubated successfully with stable vital signs at the end of the case. Prior to transfer to the neuro-intensive care unit, the patient was responding appropriately to commands and required no oxygen or hemodynamic support. She was transferred to the neuro-ICU in stable condition for recovery. The patient’s vitals on arrival to the neuro-ICU were as follows: blood pressure of 160/88 millimeters of mercury, heart rate of 111 beats per minute, temperature of 36.9 °C, and oxygen saturation of 96% on room air (Figure 1).

Two hours after arrival at the neuro-ICU, a standard one-time dose of postoperative oral nimodipine (60 mg) was given to reduce the risk of cochlear nerve dysfunction [7]. The patient’s baseline ionized calcium level prior to receiving nimodipine was 1.24 mmol/L, within the normal range. Approximately one hour after administration of nimodipine, the patient developed progressive sinus bradycardia and hypotension, refractory to rapid escalation of vasopressor support (Figure 1). Two hours after receiving nimodipine, the patient remained bradycardic and hypotensive with mean arterial pressures transduced from a right radial arterial line of less than 65 despite escalating pressor support with norepinephrine, epinephrine, and vasopressin. On physical examination, the patient was warm peripherally, euvolemic, breathing comfortably on room air with clear lung fields, and fully alert and oriented with no focal neurologic deficits. Bedside point of care ultrasound showed preserved biventricular function with no wall motion abnormalities, no evidence of right heart strain, and no significant valvular disease. Her electrocardiogram showed sinus bradycardia with no ischemic changes or conduction disease. Laboratory studies were notable for new lactic acidosis and hyperglycemia, with glucose levels increasing from a baseline of 90-132 to 165-362 mg/dL.

Given the temporal correlation between the administration of nimodipine and the onset of vasodilatory shock, the accompanying sinus bradycardia, and the lack of response to vasopressor therapy, CCB toxicity was suspected. Pharmacy and Poison Control were consulted for presumed CCB toxicity. The patient received a single dose of calcium chloride (1 g) followed by a calcium gluconate infusion (1-3 g/h) with immediate improvement in hemodynamics (Figure 1). Ionized calcium levels were serially monitored during this time and decreased from 1.98 to 1.43 mmol/L. Within several hours of initiating treatment for CCB toxicity, the patient had been successfully weaned off all pressors. Upon discontinuation of the calcium gluconate infusion, however, the patient’s profound shock returned. Calcium gluconate therapy was reinitiated, once again resulting in hemodynamic stabilization. By the conclusion of hospital day 1, the patient had been successfully weaned off all pressors and calcium gluconate therapy.

## Discussion

This report presents a rare case of CCB toxicity secondary to a single dose of oral nimodipine.

Prophylactic use of nimodipine has shown a beneficial effect on the long-term outcome of cranial nerve function following vestibular schwannoma resection. In a recent clinical trial, the risk of postoperative hearing loss was halved in those who had received nimodipine initiated pre-operatively and then continued post-operatively [8]. Although the exact molecular mechanism underlying this benefit is not fully understood, in-vitro work has suggested that nimodipine may have anti-apoptotic effects. Specifically, in recent studies, nimodipine-treated neurons and supporting cells grown under surgery-like conditions of heat and mechanical stress had lower rates of apoptosis than did their non-treated counterparts [9,10]. The anti-apoptotic benefits of nimodipine may be due to its ability to inhibit intracellular calcium accumulation and subsequent death in neurons and glial cells [11], but additional research is needed to clarify the mechanism of action.

Regarding the optimal dosing strategy for prophylactic nimodipine, the American Heart Association and American Stroke Association have recommended that a fixed dose of nimodipine should be used to delay cerebral ischemia in subarachnoid aneurysm patients [7]. Specifically, 60 mg of enteral nimodipine every four hours has been suggested to yield optimal neuroprotective benefits [7]. In clinical practice, these guidelines are often extended to include the use of nimodipine in patients with other forms of cerebral injury, as seen in this patient. Considering the variability of nimodipine metabolism based on patient-specific factors, the adverse effects of fixed-dose nimodipine may be underrecognized.

**Figure 2:**
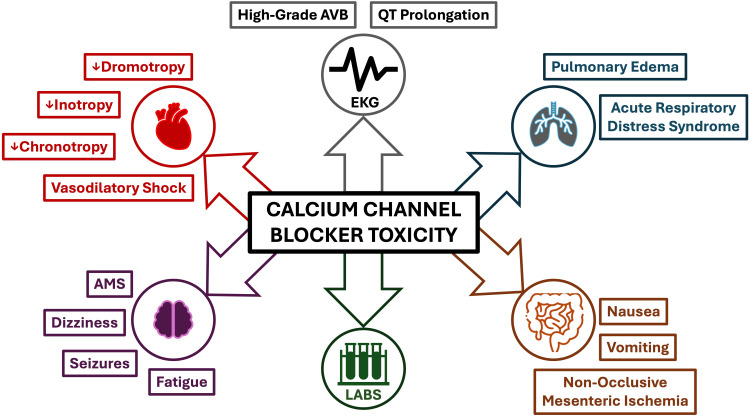
Clinical and metabolic effects of calcium channel blocker toxicity Image credits: Daniel Loriaux

Numerous studies have demonstrated that nimodipine pharmacokinetics vary as a function of genetic polymorphisms, comorbidities, age, and sex [12]. As a result, peak and steady-state plasma concentrations of nimodipine can vary by up to 10-fold [12]. Nimodipine is hepatically metabolized, and therefore, patients with severe liver disease are most susceptible to toxicity. Nimodipine toxicity causes profound vasodilatory shock by inducing negative inotropy, negative chronotropy, and peripheral vasodilation [13]. These effects of CCB toxicity are characteristically seen within one to two hours of ingestion [13]. Dihydropyridine CCBs such as nimodipine have specificity for vascular smooth muscle, and overdose of this drug group may thus present with hypotension and reflex sinus tachycardia [14]. Interestingly, this patient developed hypotension with bradycardia. This may be explained by the fact that, at high enough levels, dihydropyridine CCBs can lose their sensitivity for peripheral vasculature and subsequently block L-type calcium channels within the myocardium to evoke bradyarrhythmias [12,13,15]. Patients with CCB toxicity may also demonstrate dizziness, fatigue, altered mental status, dyspnea, and other symptoms, as highlighted in Figure 2. These symptoms can be particularly challenging to assess in the immediate perioperative setting.

The most common laboratory abnormalities in the setting of CCB toxicity are hyperglycemia and metabolic acidosis [13]. Hyperglycemia arises from CCBs inhibiting calcium-mediated insulin secretion from pancreatic Islet cells [16]. In this way, serum glucose concentration correlates directly with the degree of CCB toxicity [16]. Lactic acidosis can occur from CCB-induced inhibition of oxidative phosphorylation, a calcium-mediated process [17]. These effects of CCB toxicity were present in our patients, whose glucose levels ranged from 165 to 362 mg/dL and lactate ranged from 3.2 to 7.0 mmol/L. The hyperglycemia and lactic acidosis for the patient presented in this report quickly improved with the treatment of CCB toxicity. Early recognition of the clinical signs of CCB toxicity is essential to facilitate effective treatment (Figure 2).

The patient presented in this case had numerous risk factors for developing CCB toxicity, including reduced drug metabolism due to underlying cirrhosis and potential drug-drug interactions with anesthetic agents used during her surgery. Patients with end-stage liver disease demonstrate impaired hepatic function and a subsequent reduction in the activity of drug-metabolizing CYP450 enzymes, most notably CYP1A and CYP3A [6]. This patient received aprepitant and propofol pre- and intraoperatively, which are both known inhibitors of CYP3A4 [18,19]. Aprepitant has specifically been found to have a dose-dependent effect on CYP3A4 inhibition [19]. Genetic polymorphisms in the CYP3A gene can also alter whether a patient is an extensive, normal, intermediate, or poor metabolizer. Patients with the CYP3A5 genotype have been found to be poor metabolizers [20]. A cytochrome P450 panel was sent for our patient, and she was found to have an intermediate CYP3A5 phenotype, which also likely contributed to her risk for nimodipine toxicity [20].

Once CCB toxicity was suspected, the patient was appropriately treated with a multimodal approach in line with best practice [15], which included catecholamines, high insulin euglycemia therapy, and calcium gluconate. Following the resolution of vasodilatory shock with initial treatment for CCB toxicity, the patient presented in this case developed recurrent vasodilatory shock upon discontinuation of her calcium gluconate infusion. This second episode of hypotension reflects the short duration of action of IV calcium gluconate (30-60 minutes) and high circulating levels of unmetabolized nimodipine.

## Conclusions

The primary objective of this case report is to highlight the unique pharmacokinetic properties of nimodipine and the increased risk of toxicity that exists for patients with advanced liver disease and cytochrome P450 gene mutations. Due to substantial variations in nimodipine bioavailability and steady-state plasma concentrations, CCB toxicity may occur when the standard nimodipine dosing regimen is utilized for high-risk patients. When caring for patients with severe liver disease, a reduced dose of nimodipine should be considered, and additional caution must be taken to avoid concomitant use of cytochrome P450 inhibitors. Finally, for the patient with refractory shock who has recently received nimodipine, a high index of clinical suspicion must be maintained for the early identification and treatment of CCB toxicity.
